# Gas-Sensing Mechanisms and Performances of MXenes and MXene-Based Heterostructures

**DOI:** 10.3390/s23218674

**Published:** 2023-10-24

**Authors:** Riya Alice B. John, Karthikeyan Vijayan, Ni Luh Wulan Septiani, Andri Hardiansyah, A Ruban Kumar, Brian Yuliarto, Angga Hermawan

**Affiliations:** 1 School of Advanced Sciences, Vellore Institute of Technology, Vellore 632014, India; riyaalice.bjohn2018@vitstudent.ac.in (R.A.B.J.); vijayan.k2019@vitstudent.ac.in (K.V.); arubankumar@vit.ac.in (A.R.K.); 2 Research Center for Advanced Materials, National Research and Innovation Agency (BRIN), South Tangerang City 15314, Indonesia; nilu010@brin.go.id (N.L.W.S.); andri.hardiansyah@brin.go.id (A.H.); 3 Advanced Functional Materials Research Group, Institut Teknologi Bandung, Bandung 40132, Indonesia; brian@itb.ac.id; 4 Faculty of Textile Science and Technology, Shinshu University Ueda Campus, Ueda 386-8567, Japan

**Keywords:** 2D MXenes, heterostructure gas sensor, gas-sensing mechanism

## Abstract

MXenes are a class of 2D transition-metal carbides, nitrides, and carbonitrides with exceptional properties, including substantial electrical and thermal conductivities, outstanding mechanical strength, and a considerable surface area, rendering them an appealing choice for gas sensors. This manuscript provides a comprehensive analysis of heterostructures based on MXenes employed in gas-sensing applications and focuses on addressing the limited understanding of the sensor mechanisms of MXene-based heterostructures while highlighting their potential to enhance gas-sensing performance. The manuscript begins with a broad overview of gas-sensing mechanisms in both pristine materials and MXene-based heterostructures. Subsequently, it explores various features of MXene-based heterostructures, including their composites with other materials and their prospects for gas-sensing applications. Additionally, the manuscript evaluates different engineering strategies for MXenes and compares their advantages to other materials while discussing the limitations of current state-of-the-art sensors. Ultimately, this review seeks to foster collaboration and knowledge exchange within the field, facilitating the development of high-performance gas sensors based on MXenes.

## 1. Introduction

In recent years, the discovery of 2D materials has revolutionised the field of materials science and engineering [1]. Among these, MXenes have emerged as a promising class of 2D materials with diverse properties and applications. MXenes, a class of two-dimensional (2D) transition-metal carbides, nitrides, and carbonitrides, have emerged as a fascinating material family with exceptional properties, which are promising for a wide range of applications [2,3,4,5]. The story of MXenes began in 2011, when researchers at Drexel University discovered a novel method to etch and exfoliate MAX phases, layered ternary carbides, and nitrides, resulting in the creation of a new family of 2D materials [6]. For example, Ti_3_C_2_T*_x_* MXenes were obtained by selective etching of the A element (e.g., aluminium) from their corresponding MAX phases (e.g., Ti_3_AlC_2_) using hydrofluoric acid (HF) or other etchants [6].

MXenes are typically composed of transition metals, such as titanium (Ti), vanadium (V), niobium (Nb), chromium (Cr), zirconium (Zr), etc., and have the chemical formula M_n+1_X_n_T_x_, where M represents the transition metal, X represents carbon and/or nitrogen, and T symbolises surface termination groups, such as hydroxyl (–OH), fluorine (–F), or oxygen (–O) [5]. The number n is determined by the number of X layers, and can range from 1 to 4, while n+1 represents the number of transition metal layers [7]. The T groups on the surfaces of MXenes can be chemically modified, leading to tuneable surface chemistry and wettability. Due to this, MXenes exhibit a range of unique physical, chemical, electronic, electrical, and mechanical properties, including high electrical conductivity, high thermal conductivity, high mechanical strength, and large surface area. Additionally, they have excellent chemical stability, good biocompatibility, and good optical properties [8,9]. These properties make MXenes attractive candidates for various applications, such as supercapacitors, batteries, electrocatalysts, and sensors.

One of the most promising applications of MXenes is in gas sensors [10,11,12]. Gas sensors play a crucial role in many aspects of our lives, including environmental monitoring, industrial process control, and medical diagnostics [13,14]. A gas sensor is an electronic device that detects the presence of various gases in the environment and converts this information into a measurable signal [15]. The critical parameters for gas sensors include sensitivity, selectivity, response time, and stability [16,17,18]. Currently, the most common materials used for gas sensors are metal oxides such as tin oxide, zinc oxide, and titanium dioxide [19,20]. However, these materials have some limitations, including low selectivity, poor stability, and the need for high operating temperatures [19,20]. MXenes, on the other hand, have demonstrated exceptional gas-sensing properties, making them a promising candidate for replacing the current materials. For example, MXenes have exhibited high sensitivity and selectivity towards various gases, including nitrogen dioxide (NO_2_), ammonia (NH_3_), and hydrogen (H_2_) [11,21,22]. Moreover, MXenes have shown excellent stability and low power consumption, making them ideal for long-term use and portable applications. Furthermore, MXene-based heterostructures have shown significant advancements in gas-sensor performance compared to state-of-the-art technologies. A heterostructure is a material consisting of two or more different materials with different electronic properties [23,24]. The combination of MXenes with other materials in heterostructures can enhance gas-sensing properties by increasing sensitivity and selectivity. For example, MXenes can be combined with metal oxides or polymers to create heterostructures that exhibit enhanced gas-sensing performance [23,24,25]. Despite these advantages, MXenes and MXene-based-heterostructure gas sensors face several notable challenges. One key challenge lies in achieving optimal selectivity towards specific gas analytes while maintaining high sensitivity, as gas mixtures in real-world environments can be complex. Furthermore, the long-term stability and robustness of these sensors need improvement to ensure reliable and continuous operation over extended periods. Integration into practical devices and scaling-up of production processes are also areas of concern for the commercialisation of MXene-based gas sensors. Lastly, addressing issues related to the cost-effectiveness and availability of MXene materials at a larger scale is essential for widespread adoption. Overcoming these challenges is pivotal in realising the full potential of MXenes and MXene-based heterostructures in gas-sensing applications [23,25,26].

As stated above, MXenes have gained a lot of attention in the field of gas sensors, and numerous published reviews have highlighted their potential applications. Nevertheless, these reviews mainly focused on the synthesis of MXenes and their sensor performance [23,24], without delving into the fundamental understanding of the sensor mechanism. As a consequence, the MXene-gas-sensor field lacks a mutual comprehension of the sensing mechanism. To address this gap, a comprehensive review is needed to explain the gas-sensor mechanism of MXene-based heterostructures in depth. Thus, this review aims to foster mutual understanding within the field and pave the way for designing high-performance MXene-based gas sensors. Given the importance of the sensor mechanism in achieving high-performance MXene-based gas sensors, a thorough understanding of this mechanism is of utmost importance.

The present review addresses the lack of fundamental understanding of the sensor mechanism of MXene-based heterostructures for gas-sensing applications. By conducting a comprehensive analysis of the existing literature, this paper will highlight the significance of MXene-based heterostructures and their contribution to the advancement of the field. The review begins with gas-sensing mechanism in the pristine and MXene-based heterostructures, followed by an in-depth discussion of the properties of MXene-based heterostructures (such as compositing with other materials and their potential for gas-sensing applications). Furthermore, the review will examine various engineering strategies for MXenes and their potential for enhancing gas-sensing performance. This discussion will also cover the advantages of using MXenes over other materials, as well as the limitations of the current state-of-the-art sensors. Overall, the review aims to provide a comprehensive and detailed analysis of MXene-based heterostructures for gas sensing, with the aim of fostering mutual understanding in the field and paving the way for the development of high-performance MXene-based gas sensors.

## 2. Synthesis and Properties of 2D MXenes

We aim to provide a concise overview of the synthesis and primary characteristics of 2D MXenes to facilitate a better grasp of the relationship between synthesis, properties, and activity. Generally, MXenes emerge through the selective elimination of A layers from MAX phases, giving rise to two-dimensional materials that are usually composed of three or more atomic layers. These 2D materials possess distinct properties when compared to their three-dimensional (3D) precursor counterparts [27]. In their early synthesis, the primary etching agents predominantly comprised fluorine-containing compounds, such as HF, LiF+HCl, bifluoride salts, and molten salts containing fluorine. These etchants dictated the surface terminations of MXenes, yielding three primary variations: -F, -OH, and -O. However, in 2017, alternative non-fluorine etching methods, including electrochemical etching and concentrated alkaline hydrothermal etching, were introduced, resulting in the production of fluorine-free MXenes. More recently, a non-aqueous molten salt etching approach, employing Lewis acidic salts as etchants, has been documented to yield accordion-like MXenes with modifiable surface terminations.

In contrast to the stacked configuration of MXenes, single-layer MXene nanosheets exhibit superior chemical properties, such as a notable increase in specific surface area, favourable hydrophilicity, and a wealth of surface chemistry. In fact, the initial report on MXenes employed ultrasonic treatment to disassemble accordion-like MXenes into layers, albeit with limited success due to the robust bonding between these layers, resulting in low yields and impractical outcomes [28]. The process of obtaining single-layer nanosheets from accordion-like MXenes can be accomplished through appropriate delamination techniques, with the ease of this process directly influenced by the composition of surface functional groups. Furthermore, increasing the interlayer spacing of MXene flakes through ion intercalation is a common strategy for delaminating multilayered MXenes [28]. Unlike typical etchants like LiF+HCl, which only include metal cations without additional intercalators, the use of intercalation agents such as organic intercalation, e.g., tetrabutylammonium hydroxide (TBAOH) and dimethyl sulphoxide (DMSO) and inorganic cation intercalation, e.g., Li^+^ and Na^+^, have also proven effective in delaminating multilayered MXenes into monolayer MXenes. For readers interested in a comprehensive understanding of fundamental synthesis, we recommend referring to comprehensive reviews authored by Salim et al. [27], Naguib et al. [29], Wei et al. [28], and Lim et al. [30].

Following this, depending on the synthetic route, the produced MXenes can have significantly different properties because these properties rely on the functional groups, defects, interlayer structures, etc. MXenes are versatile materials that fulfil the essential requirements for fully functional gas-sensing devices [31]. Herein, we highlight key properties of MXenes in relation to their gas-sensing capabilities. Delaminated MXenes exhibit remarkable mechanical properties, particularly in the form of monolayers. The Young’s modulus of a Ti_3_C_2_T_x_ monolayer, for instance, approaches 333 ± 30 GPa, demonstrating their exceptional strength. Nitride-based MXenes generally possess even higher Young’s modulus values compared to carbides, and surface terminations also influence their stiffness. O-terminated MXenes are stiffer than F- and OH-terminated MXenes due to their shorter in-plane lattice constant. Additionally, the mass of the transition metal affects stiffness, with heavier metals leading to stiffer MXenes. MXenes exhibit high electronic conductivity, attributed to metallic bonding. Ti_3_C_2_T_x_ MXenes, for instance, display electrical conductivities ranging from 850 to 9880 Scm^−1^, making them among the most conductive MXenes. Conductivity varies due to factors like crystal defects, surface-terminated groups, layer spacing, and delamination. Nitride-based MXenes tend to display more metallic properties than carbide-based ones, owing to the higher electron content in nitrogen. These properties can be modulated through functional-group modifications and solid-solution formation, and some MXenes can exhibit narrow band gaps due to electron withdrawal from the transition metal to surface groups.

MXenes offer high transparency and light absorbance, with Ti_3_C_2_T_x_ MXenes in the range of 1–2 nm thickness achieving up to 91.2% optical transparency and excellent light-to-heat conversion efficiency. Different terminations impact their optical properties, with -F and -OH groups reducing visible light absorption and reflectivity but enhancing reflectivity in the UV region. They demonstrate excellent thermal stability, with Ti_3_C_2_T_x_ (T = F, OH) remaining stable up to 500 °C, or 800 °C in argon atmosphere. Surface functionalisation with functional groups helps mitigate surface oxidation. Moreover, MXenes possess high thermal conductivity, making them suitable for self-heating gas-sensing devices and aiding in heat dissipation in electronics. Hf_2_CO_2_, for instance, exhibits a room-temperature thermal conductivity of 86.25 W m^−1^ K^−1^, increasing with flake thickness. N-doped single-layer Mo_2_C MXene also shows promising thermal conductivity. There are available reviews that focus on the progress of MXene properties, which interested readers can explore [31,32,33,34].

## 3. Fundamental Sensing Mechanisms in 2D MXenes and MXene Heterostructures

The sensing mechanism within MXene structures distinguishes itself from that of metal oxides and exhibits a higher level of complexity compared to the surface adsorption or charge transfer commonly observed in traditional 2D materials. In metal-oxide-based sensors, the sensing mechanism is well-established as relying on the interactions between gas molecules and pre-adsorbed oxygen species at the surface [35]. However, the case is different for MXenes.

The fundamental sensing mechanisms inherent to 2D MXenes and MXene heterostructures play a pivotal role in their gas-sensing capabilities. While the gas-sensing mechanism of pristine 2D MXenes can be intuitively explored through computational simulations, MXene heterostructures introduce unique attributes. These attributes, including electronic structures and adsorption models, give rise to a distinct gas-sensing mechanism. Consequently, this section undertakes a comparative analysis of the gas-sensing mechanisms in both pristine 2D MXenes and MXene heterostructures, offering an in-depth elucidation of the contributing factors that shape their sensing performance.

### 3.1. Sensing Mechanism in Pristine 2D MXenes

Among the MXene family, Ti_3_C_2_T*_x_* stands out as the most extensively explored member for its applications in gas sensors. The behaviour of a Ti_3_C_2_T*_x_* sensor typically resembles that of p-type semiconductors, a trait attributed to the presence of functional groups such as −F, −OH, and −O. In the realm of gas sensing, the transfer of charge carriers is governed by two primary mechanisms: physisorption and chemisorption. At ambient temperatures, physisorption takes precedence, involving the gas molecules’ physisorption onto the surface, circumventing the participation of adsorbed oxygen species. Consequently, alterations in the electrical signal stem from the adsorption and subsequent desorption processes.

The nature of gas analytes significantly influences these changes in the electrical signal. For instance, highly reducing gases elevate electrical resistance, whereas highly oxidising gases lead to its decline. To illustrate, consider the research conducted by Kim et al. [36]. When Ti_3_C_2_ MXene encountered ammonia, the electrons generated will get combined with the holes in MXene, which decreases the majority charge-carrier concentration and thereby increases the resistance of Ti_3_C_2_T_x_ sensors. The reaction mechanism of p-type Ti_3_C_2_T_x_ MXene and reducing gas is depicted in Equations (1) and (2).

2NH_3_ + 3O^−^→N_2_ + 3H_2_O + 3e^−^ (1)

NH_3_ + OH^−^→NH_2_ + H_2_O + e^−^ (2)

This scenario mirrors the behaviour observed in V_2_CT*_x_* MXenes. However, in the case of the V_2_CT*_x_* sensor exposed to oxidising gases like NO_2_, the dynamic changes are distinct. NO_2_ molecules attach to the active sites of V_2_CT*_x_* through surface terminations like single-bonded O and single-bonded OH. Consequently, a transfer of electrons from V_2_CT*_x_* to NO_2_ molecules transpires, augmenting hole concentration within V_2_CT*_x_* (Equations (3)–(5)). This, in turn, amplifies conductivity and diminishes resistance in the V_2_CT*_x_* sensor. Furthermore, the reaction between NO_2_ and O^2−^ yields NO^2−^, consuming electrons and thereby reducing the resistance of the V_2_CT*_x_* sensor [22]. This intricate process can be encapsulated in the following equations.

O_2 (ads)_ + e^−^ → O_2_^−^, (at RT) (3)

NO_2 (gas)_ + e^−^ → NO_2_^−^ _(ads)_ (4)

2NO_2(g)_ + O_2_^−^ _(ads)_ + e^−^ → 2NO_2_^−^ _(ads)_ + O_2_ (5)

**Figure 1:**
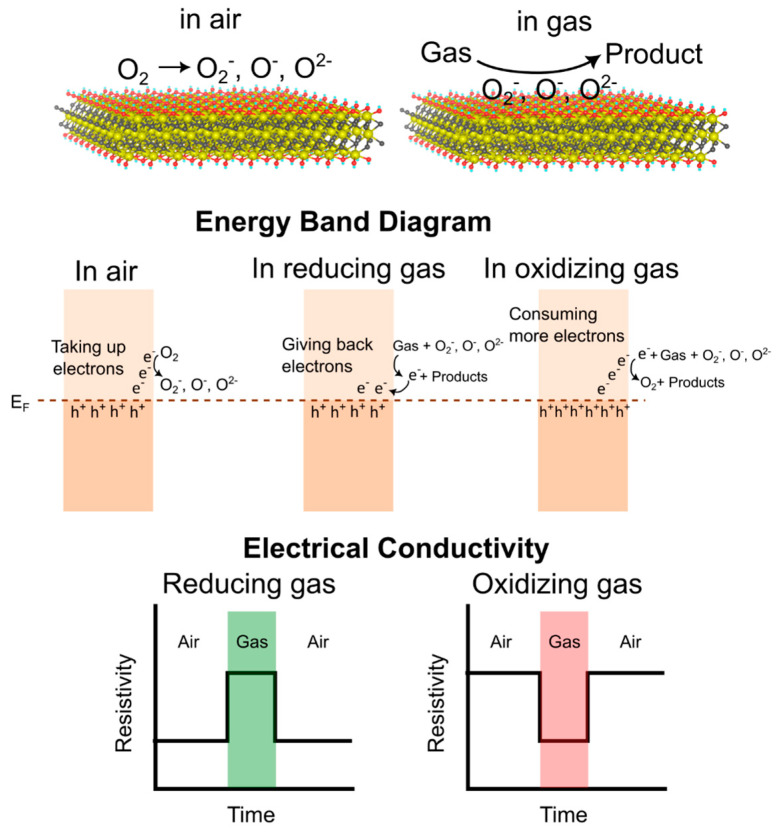
Gas sensing mechanism of the pristine 2D MXenes and the corresponding energy band diagram and electrical conductivity changes in reducing and oxidising gas.

Based on the results of these experimental studies, the gas-sensing mechanism in pristine MXenes can be comprehensively depicted in a general model, as illustrated in Figure 1. This model effectively illustrates the intricate interplay between molecular interactions and the surface of MXenes, and how these interactions relate to changes in electrical resistance and the band diagram. In this context, when oxygen molecules from the surrounding air come into contact with the MXene surface, they become adsorbed and transform into distinct species based on the operational temperature. Specifically, they assume the form of O_2_^−^ at temperatures below 150 °C, O^−^ at 150–250 °C, and O^2−^ at temperatures exceeding 250 °C. This transformation involves the oxygen molecules drawing electrons from the MXene surface. Because of this electron deficiency, MXenes that exhibit p-type conductivity will display low resistivity. Notably, the situation might differ if the MXene possesses a metal-like or n-type conductivity.

Typically, MXene-based gas sensors function at room temperature to prevent surface oxidation. As a result, the most prevalent adsorbed oxygen ions are of the O_2_^−^ type. When external gas is introduced, the reaction between the adsorbed O_2_^−^ ions on the surface and the gas leads to changes in the conductivity of the MXenes. In the presence of a reducing gas, the above-mentioned reaction results in the return of electrons to the MXenes, generating both holes and electron recombination. This phenomenon leads to an increase in resistivity due to the depletion of holes. Conversely, when an oxidising gas is present, the reaction consumes more electrons, causing an accumulation of holes. Consequently, this accumulation causes a decrease in resistivity. While this model effectively explains the gas-sensing mechanism in most pristine MXenes, it is important to note that variations could arise if the surface functional groups are altered. Take the case of APTES-functionalised Nb_2_CT_x_ MXene, for instance. Here, APTES acts as an electron acceptor, leading to a transfer of electrons from Nb_2_CT_x_ to APTES. This process establishes a new equilibrium when combined with the transfer of an electron from APTES to NO_2_. Consequently, the result is an increased resistance of the sensor, exhibiting n-type behaviour [37].

### 3.2. Sensing Mechanism in 2D MXene Heterostructures

The gas-sensing mechanism in MXene heterostructures is inherently more complex due to their ability to be combined with various types of materials, such as metal oxides, polymers, metal nanoparticles, and others. This combination leads to diverse configurations in the energy-band diagram when these components come into contact, interacting with target molecules of either reducing or oxidising gases. This complexity is further heightened when heterostructures consist of more than two materials, introducing additional challenges and uncertainties into the gas-sensing mechanism. For example, MXenes with p-type conductivity can be integrated with n-type or p-type semiconductors, resulting in the formation of p–p or n–p junctions, depending on the arrangement within the sensor device. Establishing a comprehensive model for the gas-sensing mechanism and the configuration of energy-band alignment requires addressing these complexities. Numerous studies have aimed to elucidate the gas-sensing mechanism within metal oxides/Ti_3_C_2_T_x_ heterostructures in order to develop a generalised framework.

**Figure 2:**
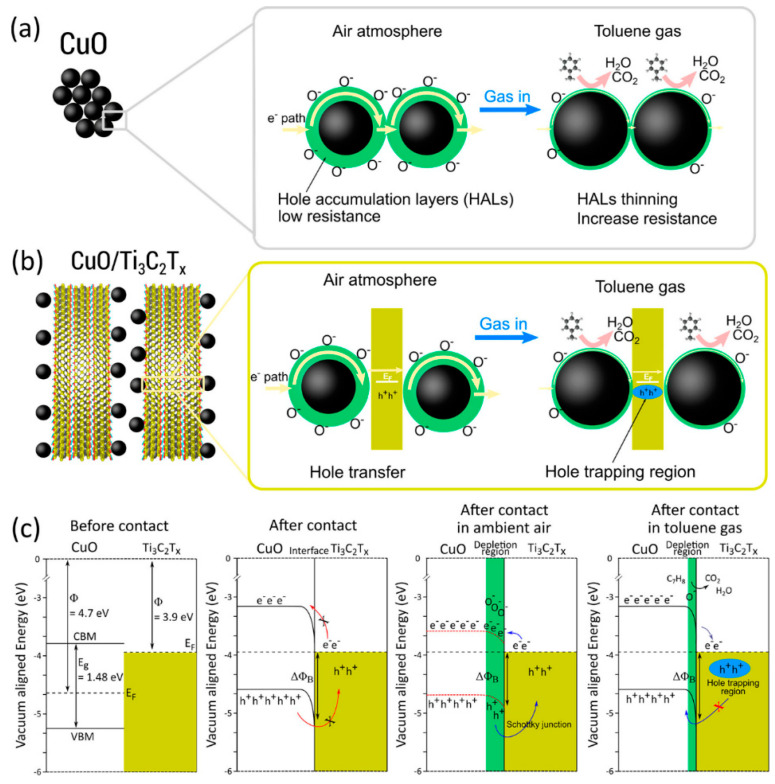
Gas sensing mechanism of (**a**) pristine CuO nanoparticles and (**b**) CuO nanoparticles/Ti_3_C_2_T*_x_* MXene hybrid heterostructures. (**c**) Band-structure alignment of CuO/Ti_3_C_2_T*_x_* before contact, after contact, in ambient air, and in toluene gas. [38]. Red color in arrow and X mean the mechanism will not occur.

Hermawan et al. [38] fabricated a sensor device consisting of p-type CuO semiconductor with metallic Ti_3_C_2_OH_2_ and found that the work function played a significant role in regulating the gas-sensing mechanism, charge transfer, and energy-band alignment. The Ti_3_C_2_T*_x_* MXene with –OH termination exhibits a work function of around 3.9 eV, lower than CuO’s work function of 4.7 eV, creating a Schottky barrier at their interface and aligning the Fermi energy levels. Charge transfer occurs bidirectionally between metal and semiconductor across the interface, limited by the barrier height ΔΦB, resulting in high room-temperature resistivity. During the gas-sensing mechanism, O^−^ ion adsorption reduces interface band bending, aiding charge transfer with the temperature rise. The Schottky barrier leads to poorer mobility in p-type/metallic CuO/Ti_3_C_2_T*_x_* than in p-type CuO, explaining the slightly higher resistance despite Ti_3_C_2_T*_x_*’s better conductivity. O^−^ removal by reducing gas thins the depletion region (HALs), raises band bending, and reinstates the Schottky barrier, causing hole trapping in Ti_3_C_2_T*_x_* (See Figure 2). One thing which should be highlighted is that Schottky hole trapping may occur if the work function is lower than the tandem p-type materials. The Schottky barrier also forms when metallic Ti_3_C_2_T*_x_* is combined with an n-type semiconductor such as W_18_O_49_ and SnO_2_ [39,40,41], regardless of the type of target gas. We have noted that, in most reported experiments, shifts in electrical conductivity tend to mirror the inherent characteristics of the paired oxides. For instance, the resistivity of n-type paired MXenes decreases and increases upon encountering reducing and oxidising gases, respectively. Therefore, the gas-sensing mechanism trends will largely align with those of the paired oxides.

**Figure 3:**
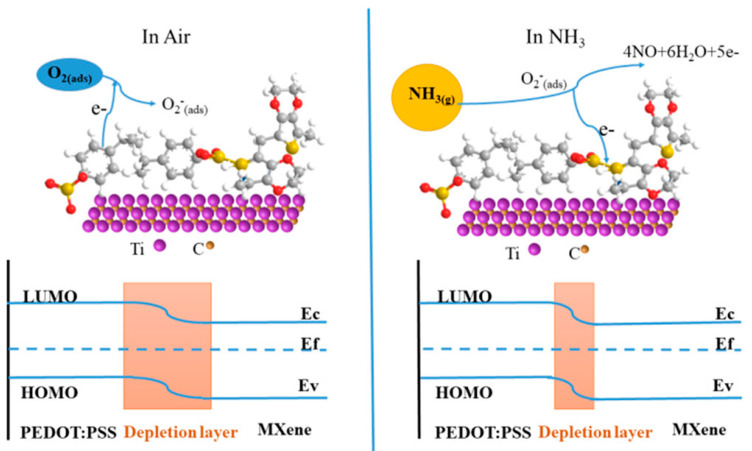
Gas sensing mechanism in Ti_3_C_2_T*_x_* MXene/PEDOT:PSS heterostructures.

Now, turning to the discussion of the gas-sensing mechanism in MXene/non-oxide heterostructures, it is worth noting that conducting polymers also exhibit n-type or p-type conductivity. The energy levels of the lowest unoccupied molecular orbital (LUMO) and the highest occupied molecular orbital (HOMO) in polymers, whether they act as donors or acceptors, are crucial factors. Given this, the gas-sensing mechanism in MXene-polymer heterostructures is likely to bear similarity to that observed in MXene/metal-oxide heterostructures. PEDOT:PSS is categorised as a donor (n-type) conjugated polymer. The formation of a band depletion stems from the absorption and ionisation of oxygen molecules, achieved by capturing electrons from the conduction band of PEDOT:PSS/MXene heterostructures. This depletion results in elevated sensor resistances. Upon exposure to reducing gas, the depletion thins, permitting electron flow and leading to a decrease in electrical resistance (Figure 3). In the case of MXene/polymer heterostructures with acceptor (p-type) conjugated polymers, the electrical resistivity of the sensors would demonstrate the opposite behaviour. However, the incorporation of MXenes in p-type polymer nanocomposites for gas-sensor applications has not been reported thus far.

Interestingly, in additional MXene composites, such as those involving carbonaceous or chalcogenide materials, the gas-sensing mechanism is similarly governed by the band-gap and work-function interactions between MXenes and the accompanying materials. In particular to the number of captured electrons, when the molecules of the adsorbed gas species are less numerous than the available electrons on the sensor surface, the sensing behaviours are influenced by the quantity of adsorbed gas species. Conversely, in situations of an abundance of adsorbed gas molecules, the sensing behaviours are determined by the electron count. In this scenario, possessing a smaller initial electron conduction volume/concentration becomes advantageous for heightened sensing, as the relative change in electron concentration due to the adsorption/desorption of the target gas becomes more pronounced. In such cases, the nature of the sensing conductivity aligns with the behaviour of the tandem materials rather than the MXenes themselves. Across all MXene-based sensor heterostructures, MXenes function as charge-carrier-trapping sites from the tandem materials during the sensing process.

The stability of MXenes is of paramount significance in the context of device manufacturing, given that the sensors in development are exposed to various and ever-changing environmental conditions. Nevertheless, prolonged resistance to oxidation at room or elevated temperatures is exceedingly inadequate for Ti_3_C_2_T*_x_* aqueous solutions, causing titanium carbide to transform into its oxidized state (TiO_2_), consequently impacting its electronic attributes. An in-depth inquiry[JY1] has revealed that MXenes can be preserved for extended periods at lower temperatures, in conjunction with certain solvents such as isopropyl alcohol (IPA), ethanol, and similar compounds. Lipatov and his collaborators engineered field-effect transistors (FETs) using single-layered Ti_3_C_2_T*_x_* flakes as conductive pathways to investigate the electronic characteristics and environmental durability of Ti_3_C_2_T*_x_* [42]. The findings unveiled a field-effect electron mobility of 2.6 ± 0.7 cm2V1s1 and a resistivity of 2.31 μΩm (equivalent to 4600 Scm^-1^) for single-layered Ti_3_C_2_T*_x_* flakes, which is an order of magnitude greater than that of the bulk Ti_3_C_2_T*_x_*. The data regarding environmental robustness indicated that Ti_3_C_2_T*_x_* FETs remain steadfast and remarkably conductive even following prolonged exposure to humid air for a duration of 70 hours. Reports have suggested that the interlayer spacing in multi-layered MXenes can be manipulated through the introduction of K^+^ and Mg^2+^ ions, thereby heightening the absorption of target gases and volatile organic compounds (VOCs). Muckley and his team [43] documented the intercalation of K^+^ and Mg^2+^ions into MXene assemblies, resulting in an augmentation of the c-lattice parameter, which dictates the extent of H_2_O adsorption in the context of humidity sensing. The durability of MXenes can be assessed through the use of Raman spectroscopy, enabling the direct observation of the oxidation process. This is accomplished by monitoring the Raman shift in the G and D spectral bands, especially the emergence of graphene bands. These spectral features indicate disruptions in the MXene lattice structure and the oxidation of MXenes [44].

**Figure 4:**
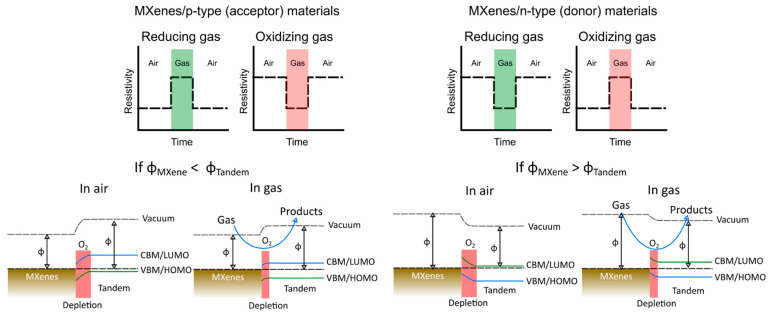
Gas sensing mechanism in MXene heterostructures; electrical response to different gas and band-energy alignment.

Upon careful consideration of the multitude of empirical data available in many reports, we elucidate the intricate gas-sensing mechanism inherent within the MXene heterostructures. This mechanism manifests through electrical responses and the consequential alignment of band energies, as visually depicted in Figure 4. Notably, the nature of the electrical response, signifying alterations in resistivity or current flow, is intrinsically tied to the inherent properties of the paired materials constituting the tandem structure. Moreover, the band-energy alignment during contact is a pivotal aspect of this sensing mechanism. This alignment, crucially influenced by the work function, serves as a critical determinant of the ensuing gas–surface interactions. Specifically, it is noteworthy that band-depletion events correspondingly coincide with the presence of adsorbed oxygen species, a phenomenon of profound significance in the context of gas-sensing mechanisms within MXenes heterostructures.

To elucidate the gas-sensing mechanism in MXene heterostructures, one can consider the following steps:

1. Determine the conductivity (metallic, p-type, or n-type), work functions, as well as the band configuration of pristine MXenes and tandem materials using standard measurements.
2. Illustrate the potential energy-band diagram alignment before and after contact, and depict the potential band bending resulting from Fermi level equalisation.
3. Conduct gas-sensing evaluations under both reducing and oxidising atmospheres, and observe the resultant change in the resistance of the MXene heterostructures.
4. Illustrate the possible charge-carrier transfer (holes or electrons) at the interface of the heterostructures during oxygen adsorption and gas exposure, and relate to the change of electrical resistance.

## 4. Engineering Approach to Enhance 2D MXene Sensing Performances

Despite displaying notable sensing properties, the MXene’s stability is compromised and it deteriorates under oxidising or humid conditions, attributable to its hydrophilic nature arising from the existence of terminal groups [45,46]. This predicament is a critical concern that necessitates addressing for the development and widespread adoption of MXene-based gas sensors in the global market. The current trends in gas-sensor technology heavily emphasise the engineering aspects of MXene, encompassing surface functionalisation and morphological tuning, among others [47]. In this discussion, we delve into two highly effective engineering strategies aimed at optimising the sensing capabilities of 2D MXenes.

### 4.1. Surface Functionalisation

**Figure 5:**
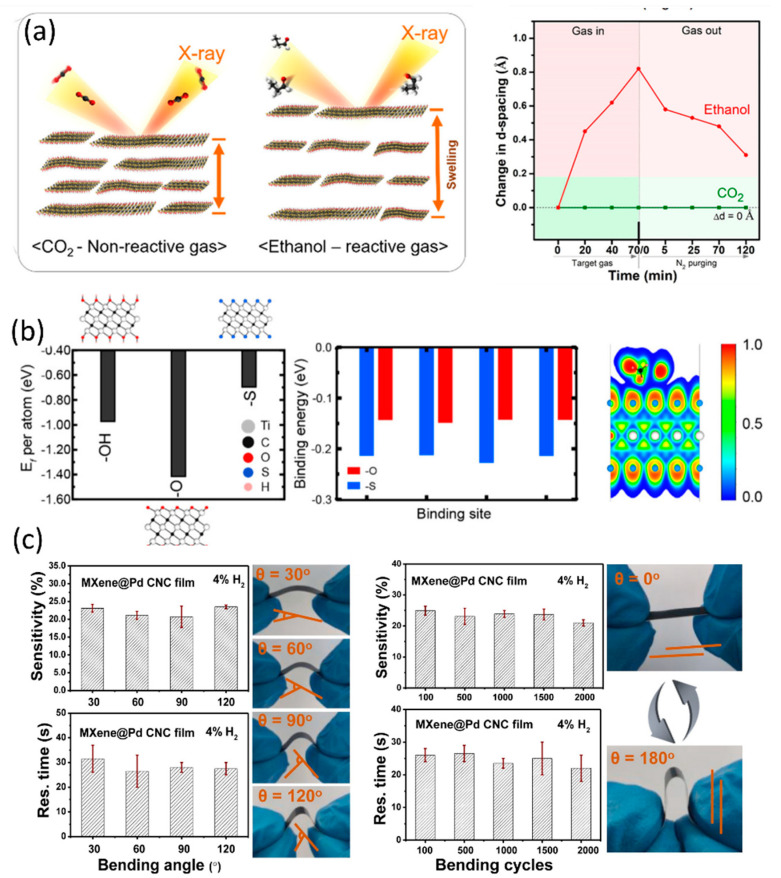
(**a**) Schematic of in situ XRD measurements of Ti_3_C_2_T*_x_* films (transferred from 0.3 mM NaOH solution) upon gas introduction and summarised (002) peak shift of Ti_3_C_2_T*_x_* films upon CO_2_ and ethanol introduction [48]. (**b**) Formation energy per atom for finding the preferential functional group of the MXenes, horizontal binding energy of toluene to titanium carbide MXenes with −O and −S as the surface termination group at four different sites, and ELF indicating variation in the charge distribution of S atoms around the adsorbed molecule [49]. (**c**) Sensitivities and response times of MXene@Pd CNC film sensor to 4% H2 under different bending angles and after n-time bending cycles and one bending cycle show from θ = 0° to 180° and back to 0° [53].

In recent years, researchers have explored various approaches to functionalise pure MXenes and enhance their surface properties and reactivity. Among these approaches are ion intercalation using K^+^ and Mg^2+^ ions, decoration with noble metals, fluoroalkyl-silane treatment, and transition-metal oxyfluoride modification, to name a few [45,47,48,49]. By improving the VOC-sensing attributes of MXenes, functionalisation has opened up exciting possibilities for these materials in diverse applications. Notably, functionalisation using nanostructures and ion intercalation has led to significant improvements in sensor performance, as reflected in Table 1. Furthermore, ion intercalation serves as an alternative to overcome the limitation of MXenes due to their highly conductive nature and to improve sensor characteristics. For example, Yang et al. [50] found that intercalation of Na^+^ into MXene increased the N–Ti sites and O/F ratio, resulting in a marked enhancement of sensor response to NH_3_. The proposed work achieved high-humidity sensing attributes and 28.87% response for 100 ppm NH_3_ gas. Muckley et al. [51] demonstrated that intercalation of K^+^ and Mg^2+^ ions in MXenes causes spacing between MXene sheets, leading to trapping of water molecules and an increase in c-lattice parameters. Koh et al. [48] investigated Na^+^ intercalation in MXenes to analyse interlayer swelling for the sensing of CO_2_ and C_2_H_6_O via in situ XRD measurements. The work assessed the sensor performance under various NaOH concentrations and obtained a 9.99% response for 0.1% C_2_H_6_O for 0.3mM NaOH (Figure 5a). Shuvo et al. [49] proposed sulfur(S)-functionalised MXenes that showed appreciable sensing parameters towards various VOCs, particularly for toluene detection at the parts per billion level [49]. S-doping enhanced the spacing between the layers of Ti_3_C_2_T*_x_* (from 0.96 to 1.91 nm) for the (002) plane. This increases the surface-to-volume ratio and thereby the analyte interaction. Moreover, the electronegative S ions extract electrons from MXene and vary the sensor current and response of 79.5% to toluene at room temperature (Figure 5b). In addition, Chen et al. [47] presented a novel method of fluoroalkylsilane (FOTS) intercalation with Ti_3_C_2_T*_x_* to increase the oxidative stability of MXene. The Ti_3_C_2_T*_x_*-F displayed an enhanced surface area and highest response of 14% to ethanol gas due to the enhanced interspacing.

Noble metal decoration is another effective methodology for improving the sensor characteristics of MXenes. For instance, Li et al. [52] suggested Ag-modified MXenes as humidity sensors with a polymer adhesive layer. The novel approach increased the ionic conductivity and thereby introduced a flexible sensor with a high sensitivity of 106%, which records the fluctuations in humidity with the change in human voices and motion of hands, which shows its potential for application in robotics. Zhu et al. [53] reported a novel hydrogen sensor by decorating Pd on MXene. The sensor exhibits a fast response time of (32 ± 7) s and an appreciable sensitivity (23.0 ± 4.0), and the highest adsorption rate is due to the induced doping of electrons by Pd nano-clusters (Figure 5c). Zou et al. [54] proposed composite Fe_2_(MoO_4_)_3_/MXene nanocomposites for the effective detection of n-butanol. Kumar et al. [37] revealed an amine-functionalised MXene for effective sensing of NO_2_ gas detection. Wang et al. [55] suggested intercalation of TiOF_2_ for the surface functionalisation of MXene for a humidity sensor. The presence of TiOF_2_, which has a larger band gap, catalytic behaviour, and a capability of adsorbing hydrophilic groups, advances the specific surface area and stability of MXene and stabilises the terminal groups, thereby enhancing the sensitivity and selectivity of MXene towards humidity.

Surface functionalisation with various strategies has been found to increase the adsorption rates and sensing capability of MXenes for various analytes. The detailed analysis reflected that the sensor characteristics of pure MXenes are enhanced by surface functionalisation with strategies such as ion intercalation, doping with sulfur or noble metals, and modification of terminal groups. Overall, the diverse functionalisation techniques have the potential to expand the range of applications for MXene-based sensors.

**Table 1:** Comparative study on sensor parameters of surface functionalised and layered MXene-based compositions.

Material	Target Gas	T	Response Time (s)	Recov. Time (s)	Conc.	Response (R_a_/R_g_) or (R_g_/R_a_)	Refs.
Alkalised organ-like MXene	NH_3_	RT	-	-	100 ppm	28.87	[50]
Ti_3_C_2_-Mg and Ti_3_C_2_-K	H_2_O	RT	-	-	-	0.8	[51]
NaOH-treated Ti_3_C_2_T_x_	CO_2_ and C_2_H_6_O	RT	-	-	1 %	9.995	[48]
S- Ti_3_C_2_T_x_	C_7_H_8_	RT	-	-	50 ppm	79.5	[49]
Ti_3_C_2_T_x_-F	C_2_H_6_O	RT	-	-	30 ppm	14	[47]
Ti_3_C_2_/Ag	H_2_O	RT	80 ms	-	-	106 800%	[52]
Ti_3_C_2_T_x_ MXene@Pd	H_2_	RT	-	-	-	40	[53]
Fe_2_(MoO_4_)_3_/MXene	C_4_H_8_O and C_8_H_10_	120 °C	18	24	100 ppm	43.1 and 39.5	[54]
Ni(OH)_2_/Ti_3_C_2_T_x_	NH_3_	RT	-	-	10 ppm	6.2	[56]
Nb_2_CT_x_ MXene	NO_2_	RT	-	-	25 ppm	31.52	[37]
TiOF_2_@Ti_3_C_2_T_x_	H_2_O	RT	16	20	-	993	[55]

### 4.2. Layering Structures

With regards to their functional attributes, MXenes are prospective materials for gas-sensing applications. However, the stacked sheets of MXene can lead to reduced specific surface areas, thereby diminishing the sensing performance, as evidenced by previous studies [25,57]. The influence of 2D layered materials is particularly noteworthy because of their diverse benefits, including a significant surface-to-volume ratio, exceptional flexibility, modifiable electronic structure, and remarkable mechanical stability [58]. The etching technique used for structuring MXene from the MAX phase typically leads to the development of layered structures. To achieve mono-layered MXene, an additional exfoliation approach is necessary, usually followed by mechanical shaking or sonication [59]. Delamination or intercalation allows for the exploration of exclusive characteristics of 2D materials. Mashtalir et al. demonstrated the intercalation of organic molecules into the layers of MXene structures, resulting in the intercalation of f-Ti_2_C_2_. This work involved the intercalation of (CH_3_)_2_SO, which aided the delamination of the f-Ti_3_C_2_ layers into discrete 2D sheets of MXene.

The appropriate selection of the intercalant, along with the parameters used for the process, significantly impact the sensing and functional attributes of the material. The most commonly reported intercalants include dimethyl sulfoxide, isopropyl amine, urea, and hydrazine [60,61,62,63]. Mashtalir et al. investigated the effects of hydrazine intercalation on the characteristics of MXene and found a decrease in surface groups, resulting in surface modification. Additionally, the intercalation increased the number of active sites on the surface, leading to improved adsorption and sensor parameters of the material [63]. Another crucial property of MXenes as layered structures in gas sensors is the thickness of the sensor material. Therefore, thinner MXene layers exhibit a higher sensor response due to the active sites available in the exposed MXene-based sensor [64].

## 5. Two-Dimensional MXene-Based Heterostructures as High-Performance Gas-Sensing Materials

Recent investigations have substantiated that MXenes boast an extensive surface area, exceptional electrical conductivity, and a multitude of functional groups. Despite these advantages, the metallic nature of MXenes, their poor stability, narrow band gap, and low detection limit have been identified as major shortcomings of these materials in their role as gas-sensing elements. Recent advances have sought to address these issues by utilising MXene-based composites with diverse materials such as metal oxide, chalcogenides, and carbonaceous materials, to name a few. These composite materials have been shown to offer improved adsorption sites, defects, and sensing characteristics. In this section, we elucidate the pertinent findings reported in the existing literature on gas sensors that are based on MXene-nanocomposites.

### 5.1. MXene/Metal-Oxide Heterostructures

Metal-oxide semiconductors (MOS) have been widely investigated for gas sensing due to their exceptional specific surface area, facile synthesis, and high response to toxic gases [65,66,67,68]. However, MOS gas detectors have drawbacks such as cross-selectivity, low selectivity, and high working temperature [38,41,69,70,71]. Nevertheless, recent research has shown that hybridising metal oxides with MXenes can overcome these limitations and improve the performance of volatile organic compound (VOC) sensors [72]. Researchers have prepared and tested various MXene/metal-oxide composite combinations, such as Co_3_O_4_ [70,73], WO_3_ [74], TiO_2_ [71], SnO_2_ [41], and CuO [38,75] for VOC detection in breath analysers, food quality, and environmental applications [76,77]. Detailed summaries of the relevant literature are provided in Table 2.

The use of MXene/metal-oxide composites has also been explored for NH_3_ detection, with promising results. Tai et al. [71] designed a TiO_2_/Ti_3_C_2_T*_x_* film for NH_3_ detection, which showed a significantly improved sensor response and response/recovery times compared to pure MXene due to modulation of a self-built electric field. Ranjbar et al. [78] proposed a non-invasive breath analyser for early detection of chronic kidney diseases, using a marigold-flower-shaped V_2_O_5_/CuWO_4_ integrated with Ti_3_C_2_T*_x_* sheets for NH_3_ sensing. This sensor is portable, cost-effective, and has high sensitivity due to transduction in electrical resistance (Shottky junction) when exposed to air and NH_3_. Zhang et al. [75] investigated the characteristics of MXene/CuO composites for NH_3_ sensing applications, demonstrating their potential as wearable NH_3_ sensors, using a triboelectric nanogenerator and polytetrafluoroethylene for room-temperature sensing. Guo et al. [74] developed a highly sensitive NH_3_ sensor with minimised power consumption using Ti_3_C_2_T*_x_*/WO_3_ composites, which showed remarkable stability, response (24.8/100 ppm), and reproducibility, due to numerous active sites and adequate electron transportation between the heterojunction. He et al. [41] proposed an MXene/SnO_2_ composite sensor for room-temperature NH_3_ sensing, which exhibited high selectivity, fast response, and recovery time (<30 s), in exposure to NH_3_ gas, thanks to the difference in Fermi level driving the sensing mechanism, and a wireless sensor developed using inductor–capacitor antenna and a heterojunction developed using the LTCC technique.

**Figure 6:**
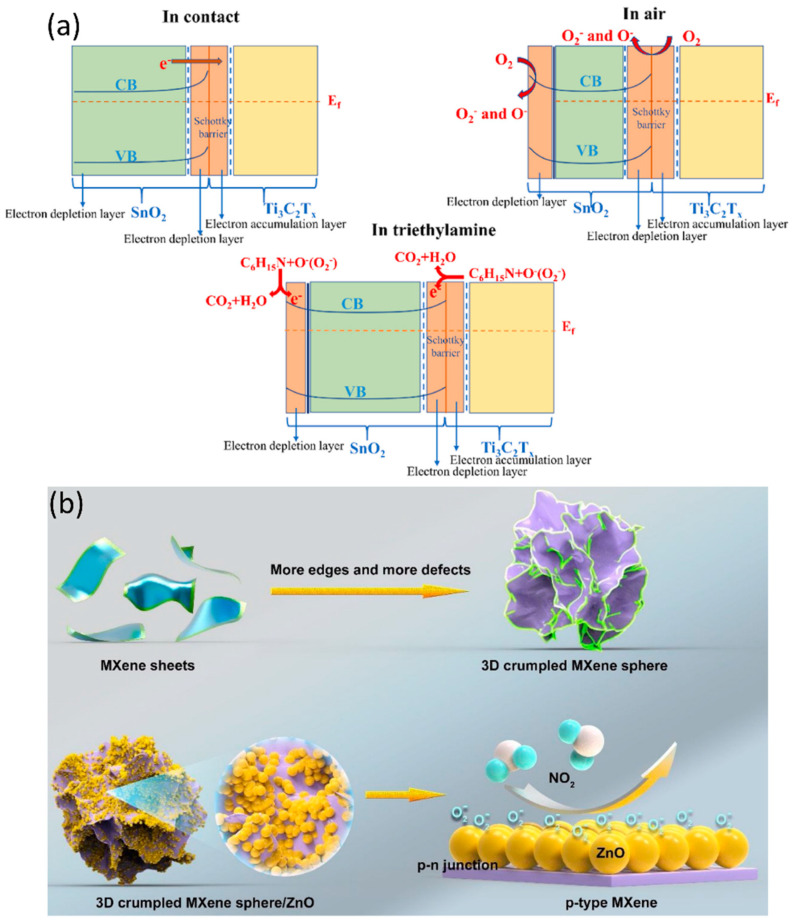
(**a**) Band−energy diagram of SnO_2_-nanosheet/Ti_3_C_2_T*_x_*-MXene nanocomposites in air and triethylamine [79]. (**b**) The schematic diagram illustrating the increase of the edges and defects of a 3D-crumpled MXene sphere and the effect of ZnO nanoparticles on NO_2_ sensing of 3D−crumpled MXene sphere/ZnO [69].

In addition, the MXene/metal-oxide composites have successfully detected various VOCs that were previously difficult to detect, including triethylamine, toluene, acetone, nitrogen dioxide, methanol, hexanal, and formaldehyde. Liang et al. [79] proposed using SnO_2_/Ti_3_C_2_T_x_ composites for detecting triethylamine gas at 140 °C. The optimised configuration and band structure of the sensor are displayed in Figure 6a. The MXene’s interconnected porous composite structure resulted in enhanced sensing attributes toward triethylamine. Hermawan et al. [38] discovered a sensor using CuO/Ti_3_C_2_T_x_ to detect toluene gas, which is the least detected gas using metal oxide to date. The work proposes a work-function matching strategy as a sensing mechanism and highlights electronic self-assembly’s impact on enhancing sensing parameters. The proposed work shows a speedy response time of 5 s and a good response of 11.4 to toluene. Sun et al. [39] developed a ppb-level acetone sensor using in situ prepared W_18_O_49_ nanorods on Ti_3_C_2_T_x_ sheets via a solvothermal procedure. The sensor exhibited enhanced sensing parameters such as fast response and recovery times, low detection limit, and a good response of 11.6 for 20 ppm of acetone gas. This improvement is attributed to the uniform distribution of W_18_O_49_ on MXene sheets, combined interfacial reactions, and the removal of –F during the synthesis route. Wang et al. [80] synthesised SnO-SnO_2_/Ti_3_C_2_T_x_ via a hydrothermal step to detect acetone gas. The Ti_3_C_2_T_x_ served as conductive sheets for carrier transportation and as a hole accumulation region when the sensor was exposed to acetone gas, and the p–n junction enhanced the response and decreased the induction temperature. Liu et al. [81] developed α-Fe_2_O_3_/Ti_3_C_2_T_x_ composites for effective sensing of acetone at room temperature. The study used DFT calculations to explain the gas-sensing mechanism behind the prepared material and acetone gas at room temperature. Zhu et al. [82] synthesised ZnO/Ti_3_C_2_T_x_ composites for effective sensing of acetone gas and obtained a response of 14.4 for 100 ppm of target gas at 320 °C, which is a six-fold response compared to pure ZnO. Furthermore, Liu et al. [71] proposed another composite of MXene with TiO_2_ and tested the sample for nitrogen dioxide (NO_2_) detection. The study is a combination of first-principle calculations and experimental tests, and the study highlights the room-temperature sensing of 5 ppm of the target gas with speedy response and recovery times. Yang et al. [69] discussed a room-temperature NO_2_ sensing approach for MXene by preparing a composite with ZnO. The p–n heterojunction, formed between the 3D-crumpled MXene sphere and ZnO, resulted in appreciable sensing attributes of the composite towards NO_2_ compared to those of 3D-crumpled MXene and ZnO tested separately towards NO_2_ (Figure 6b). Sun et al. [73] proposed and prepared Co_3_O_4_@PEI/MXene to detect NO*_x_* molecules, and the diffusion capability of the target gas and the electron transportation at the interface contributed to the enhanced sensing attributes. Wang et al. and Gasso et al. also proposed NO_2_ sensors by developing composites with ZnO and WO_3_, respectively, and their works made use of interfacial interactions for improved sensing attributes.

Nanocomposites consisting of MXene and In_2_O_3_ were investigated by Liu et al. [83] to determine their ability to sense methanol. The formation of the Schottky junction contributed to the enhanced surface area, mesoporous nature, and improved band gap (ranging from 1.18 eV to 2.08 eV). Meanwhile, Kuang et al. [84] demonstrated that Ti_3_C_2_T_x_/TiO_2_, which they synthesised and analysed structurally and morphologically, was capable of sensing various volatile organic compounds (VOCs) with a response improvement ranging from 1.5 to 12.6 times that of purely MXene-based sensors. The altered carrier density and interfacial heterojunction attributed to the heightened sensitivity. The MXene skeleton’s presence in the sensor facilitated a high signal-to-noise ratio. This, combined with the facile fabrication technique and low power consumption, rendered the sensor ideal for health monitoring purposes. Furthermore, Zhang et al. [70] developed an MXene/Co_3_O_4_ composite that senses formaldehyde at room temperature. The wearable device uses piezoelectric nanogenerator (PENG) technology to capture human motion energy. The interfacial interactions between the materials contributed to the exceptional sensing properties, including a low detection limit of 0.01 ppm and a speedy recovery time of 5 s. Lastly, Bu et al. [85] highlighted the ethanol-sensing capabilities of in situ grown Co_3_O_4_/Ti_3_C_2_T_x_ composites. The 2D Ti_3_C_2_T_x_ sheets in the composite facilitated platform nucleation and metal oxide’s rapid growth, while the porous Co_3_O_4_ provided more adsorption sites for gas sensing. The heterojunction played an essential role in augmenting the sensing properties of the proposed MXene/metal-oxide composite.

The detailed literature survey summarised the pros, cons, and gaps of MXene/metal-oxide composites for gas-sensing applications. TiO_2_, Co_3_O_4_, and SnO_2_ were the most recurrently used metal oxides as a composite with MXene for detecting target gases and it is noted in Table 2 that MXene’s composites with SnO_2_ and WO_3_ provided maximum sensor attributes for various gases. The most explored gases are ammonia and acetone, whereas ZnO showed good sensing characteristics for oxidising gases such as nitrogen dioxide. There are other reducing VOCs, particularly acetone and methanol, that were detected using α-Fe_2_O_3_ and In_2_O_3_. 

MXenes are known for their unique semiconducting properties, with p-type characteristics that make them particularly responsive to various gases. When exposed to reducing gases, these materials exhibit a positive response, causing an increase in device resistance. However, the thickness of the MXene film plays a crucial role in determining their sensing performance. As the thickness of the MXene sheets increases, the sensor's responsiveness diminishes, affecting both reducing and oxidizing gases. The sensing behavior of MXenes is also influenced by the choice of carbon precursors, such as graphite, TiC, and lampblack, as well as the size of Ti_3_C_2_T*_x_* flakes. Smaller flakes have been observed to provide superior responses due to their shorter gas diffusion paths. Ti_3_C_2_T*_x_* film serves as an excellent metallic conduit, ensuring optimal electrical conductivity, minimal noise, and enhanced signal generation, especially in the context of ethanol sensing. Remarkably, single-layered Ti_3_C_2_T*_x_* displays exceptional selectivity towards NH3 at room temperature, combined with reasonable sensitivity. This selectivity has been confirmed through first-principles calculations, emphasizing factors such as strong negative adsorption energy, charge transfer, and the smaller size of NH3 compared to that of other test gases, all contributing to the interaction and deformation of the Ti_3_C_2_O_2_ surface [36].

In a separate study, Soo-Yeon Cho et al. [86] synthesized two phases of molybdenum carbide (a-MoC_1-x_ and b-Mo_2_C) with high porosities and metallic characteristics. These unique properties stem from variations in the density of state (DOS) localizations, as revealed through DFT calculations. a-MoC_1-x_ with a nanorod morphology and b-Mo_2_C as nanoparticles exhibited extraordinary physical and chemical properties, resulting in high sensitivity to NO_2_ and a low detection limit. However, it is worth noting that MXenes are prone to oxidation, which can hinder their electronic properties during long-term storage, especially in the presence of water, leading to the conversion of titanium carbide into its oxidized form (TiO_2_). The sensing characteristics of Ti_3_C_2_T*_x_* for both reducing (CO and NH_3_) and oxidizing (NO_2_) gases at room temperature was also investigated. Stable response-recovery curves were observed for reducing and oxidizing gases, although NO_2_ sensing resulted in a significant baseline resistance drift, potentially due to the oxidation of Ti_3_C_2_T*_x_* in an oxidizing environment. The study also evaluated various volatile organic compounds (VOCs), including acetone, ethanol, and toluene, for their sensing behavior with MXenes.

A novel class of MXenes, V_4_C_3_T_x_, demonstrated effective acetone sensing at room temperature, attributed to substantial differences in adsorption behavior. MXenes with surface functional groups (–O and –OH) showed interactions with various VOCs. Mo_2_CT_x_ exhibited higher chemical activity compared to that of Ti-based MXenes, leading to increased gaseous interactions and improved sensing performance. Toluene detection with 2D Mo_2_CT_x_ yielded impressive results. Furthermore, a sensor based on V2CTx was created on a flexible polyimide substrate for the detection of nonpolar gases at room temperature. It successfully detected benzaldehyde, DNT (2,4-dinitrotoluene), and indole with low detection limits for each gas. The excellent properties of MXenes, as discussed earlier, offer a promising avenue for effective humidity sensing at room temperature. Single-layered Ti_3_C_2_T*_x_*, Ti_2_C_3_ layers, and Ti_3_C_2_, in conjunction with quartz crystal microbalances (QCMs),[JY2] have shown impressive results in relative humidity sensing. Rapid response and high sensitivity were observed in various studies involving MXenes paired with QCM for humidity detection. In Table 2, we provide an overview of the performance of pristine MXene-based sensors for gases, volatile organic compounds (VOCs), and humidity. It is noteworthy that the majority of the target gases and VOCs are of the reducing type, and these sensors exhibit remarkable detection capabilities at room temperature. Pristine MXenes, characterized by their metallic conductivity, are enriched with terminal groups, such as –OH and –F, owing to the etching process. These functional groups play a pivotal role in offering a wealth of active sites and charge transfer opportunities.

**Table 2:** Comparison of various literature studies of MXene/metal-oxide composite-based gas sensors.

Tandem Material	Target Gas	T	Response Time (s)	Recov. Time (s)	Conc.	Response (R_a_/R_g_) or (R_g_/R_a_)	Refs.
CuO	C_7_H_8_	250 °C	270 s	10 s	50 ppm	11.4	[38]
TiO_2_	NH_3_	25 °C	0.65	0.52	10 ppm	3.1	[71]
Co_3_O_4_	NO_2_	RT	1.6 s	73.1 s	100 ppm	27.9	[73]
ZnO	NO_2_	25 °C	-	-	100 ppm	41.93	[77]
Co_3_O_4_	HCHO	RT	83 s	5 s	10 ppm	9.2	[70]
W_18_O_49_	C_3_H_6_O	300 °C	5.6 s	6 s	20 ppm	11.6	[39]
WO_3_	NH_3_	RT	-	-	1 ppm	22.3%	[74]
CuO	NH_3_	RT	43 s	26 s	100 ppm	24.8	[75]
SnO_2_	NH_3_	RT	36 s	44 s	50 ppm	40	[41]
SnO-SnO_2_ (p-n junction)	C_3_H_6_O	RT	18 s	9 s	100 ppm	12.1	[80]
TiO_2_	C_6_H_14_O	25 °C	293 s	461 s	10 ppm	3.4	[84]
V_2_O_5_/CuWO_4_	NH_3_	25 °C	-	-	51 ppm	53.5	[78]
α-Fe_2_O_3_	C_3_H_6_O	RT	5 s	5 s	5 ppm	16.6	[87]
In_2_O_3_	CH_3_OH	RT	6.5 s	3.5 s	5 ppm	29.6	[83]
TiO_2_	NO_2_	RT	2	3.8	5 ppm	1.13	[88]
SnO_2_	C_6_H_15_-N	140 °C	1 s	1 s	50 ppm	33.4	[79]
ZnO	C_3_H_6_O	320 °C	8 s	12 s	100 ppm	14.4	[82]
WO_3_	NO_2_	RT	182 s	75 s	0.2 ppm	78	[89]
Co_3_O_4_	C_2_H_6_O	200 °C	50 s	45 s	50 ppm	190	[85]
ZnO	NO_2_	-	17 s	24 s	50 ppb	81	[90]

### 5.2. MXene/Polymer Heterostructures

Polymers belong to a class of materials that showcase various properties like conductivity, flexibility, sensitivity, and an array of functional groups for surface reactions, target gases, and low-temperature operating conditions. These characteristics make them ideal for chemical-sensor applications when combined with MXene [91,92,93]. A thorough review of the existing literature demonstrates that MXene/polymer composites can sense VOCs at room temperature, and this information is compiled in Table 3. MXene/polymer composites have been prepared and tested for gases such as ammonia, acetone, and methanol, making them suitable for use in various industries, healthcare, food quality monitoring, and other applications [94,95,96].

**Figure 7:**
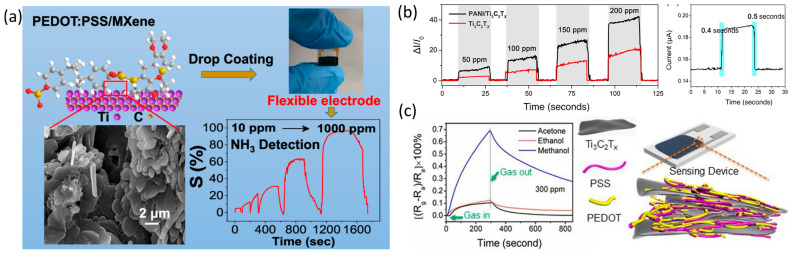
(**a**) A room-temperature ammonia (NH_3_) sensor based on PEDOT:PSS/MXene composites [97]. (**b**) Dynamic transient for response of Ti_3_C_2_T*_x_* and PANI/Ti_3_C_2_T*_x_*-based flexible sensors exposed to 50–200 ppm ethanol gases. Response and recovery times of PANI/Ti_3_C_2_T*_x_*-based flexible sensors to 150 ppm ethanol gases [91]. (**c**) A methanol gas-sensor performance based on a blend of PEDOT:PSS and Ti_3_C_2_T*_x_* [93].

While pure MXene-based NH_3_ sensors are known to have excellent sensing attributes, they are limited by their long recovery times due to the adsorption energy of NH_3_, which causes drift in the baseline resistance of the sensor. To address these limitations, researchers have developed MXene composites with polyaniline (PANI) [92], poly(styrene sulfonic acid) (PEDOT:PSS) [97], and other materials. For instance, Li et al. [98] created a flexible polymer (PANI)/MXene composite using an in situ method to develop an NH_3_ sensor with enhanced sensing characteristics compared to pure Ti_3_C_2_T_x_. The sensor showed 20–80% humidity dependence at a lower operating temperature, which makes it a viable option for agricultural applications. The sensor’s superior sensing attributes are due to Schottky-junction formation, and simulation studies have confirmed its practicality for use [98]. Similarly, Jin et al. [97] synthesised an NH_3_ sensor with an PEDOT:PSS/MXene composite using in situ polymerisation. The sensor had a fast recovery time and a good response of 95 for 1000 ppm of gas at room temperature as shown in Figure 7a. The synergistic effect of both polymer and MXene was responsible for the appreciable sensing parameters.

Zhao et al. [91] explored a novel ethanol sensor, based on a polyaniline/MXene (PANI/Ti_3_C_2_T_x_) composite, for real-time electronic sensing. The study used density functional theory (DFT) simulations and electrosensitive experiments to demonstrate the composite’s excellent electrocatalytic properties. The PANI/Ti_3_C_2_T_x_ composite showed an impressive response of 41.1% and fast response and recovery times of 0.4 s and 0.5 s, respectively, when exposed to ethanol gas at room temperature (Figure 7b). Wang et al. [93] investigated the volatile organic compound (VOC) sensing properties of a Ti_3_C_2_T_x_/poly(3,4ethylenedioxythiophene):poly(styrene sulfonate) (PEDOT:PSS) composite at room temperature. The sensor exhibited a response to various VOCs, with the highest response being to methanol due to the synergistic effect of the polymer and MXene. The sensor demonstrated a 5.54 response to methanol at room temperature, with fast response and recovery times, as depicted in Figure 7c.

Moreover, Wang et al. [99] presented a humidity sensor using poly(vinyl alcohol)/MXene (PVA/Ti_3_C_2_T_x_), and a monolayer of MoSe_2_-based piezoelectric nanogenerator (PENG) was produced using the chemical vapor deposition (CVD) technique. The sensor demonstrated a significant response (∼40), fast response/recovery time (0.9/6.3 s), and excellent repeatability. The sensor is capable of detecting moisture in human skin, highlighting its potential for real-time sensing. Li et al. [100] discussed a polymer/MXene composite-based humidity sensor as a non-invasive monitor of physiological processes. The study used Ti_3_C_2_T_x_/chitosan–quercetin layers to enhance the response of Ti_3_C_2_T_x_ to water molecules. Incorporating the polymer layer into MXene enhanced the adsorption of water molecules and contributed to the intercalation effect, resulting in an improved sensor response. Zhou et al. [101] developed a CO_2_ sensor using a nitrogen-doped Ti_3_C_2_T_x_/polyethyleneimine (PEI) composite with reduced graphene oxide (rGO). The study utilised the notable hydrophilicity of N-Ti_3_C_2_T_x_ and PEI, the reversibility of reactions between amino-enriched polymer and acidic gas molecules, and an rGO layer to support the sensor’s performance. Zhi et al. presented a flexible MXene/polyaniline/bacterial-cellulose composite [102]. Additionally, Sardhana et al. [95] demonstrated a MXene/TiO_2_/cellulose-acetate composite for the smart detection of ammonia. The hybridisation of carbon nanofibers as a substrate provided flexibility as well as an enhanced surface area. The heterojunction facilitated the adsorption/desorption ability of NH_3_ molecules. Lastly, Liu et al. [103] discussed a humidity detection sensor using an MXene/chitosan composite. The study demonstrated the sensor’s characteristics and morphological images.

MXene/polymer composites have been shown to have excellent sensing properties for various gases, VOCs, CO_2_, and humidity, exhibiting fast response and recovery times, high sensitivity, and good repeatability. The use of MXene/polymer composites in sensor technology has opened up new possibilities for the development of high-performance sensors for various applications, including real-time electronic sensing and physiological monitoring. The flexibility and enhanced surface area provided by the composites make them suitable for the development of flexible sensors that can be used for wearable technology.

### 5.3. MXene/Carbonaceous Heterostructures

Carbonaceous materials refer to any materials containing carbon, including graphite, carbon nanotubes, graphene, activated carbon, and carbon fibres, among others. These materials are known for their high thermal and electrical conductivities, making them useful in a variety of applications, such as electrodes for batteries and fuel cells, conductive additives for polymers and composites, and sensors. Graphene, for example, is considered one of the most conductive materials known, with a conductivity that is orders of magnitude higher than copper. Carbon nanotubes and carbon fibres are also highly conductive and are used in applications where strength and conductivity are required, such as in the aerospace and automotive industries. Activated carbon, on the other hand, is highly porous and is commonly used for adsorption and purification processes due to its large surface area and ability to selectively adsorb certain molecules. 

**Figure 8:**
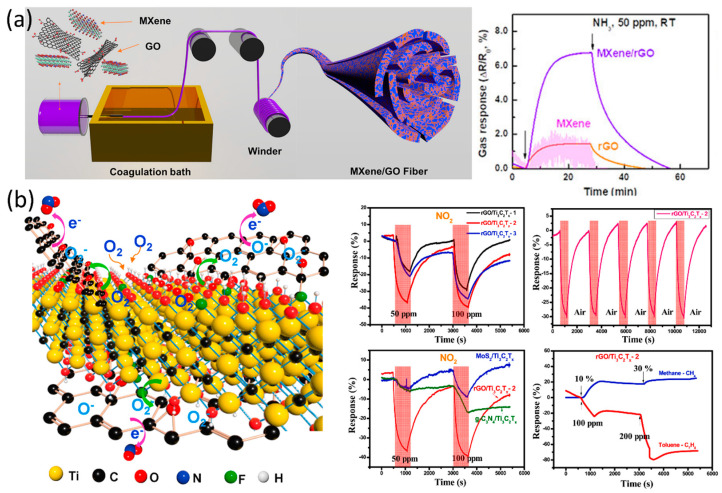
(**a**) Schematic illustration of the spinning process for MXene/GO hybrid fibre, and the corresponding gas response of MXene film, rGO fiber, and MXene/rGO hybrid fibre (40 wt % MXene) [106]. (**b**) Schematic illustration of NO_2_-sensing mechanism of rGO/Ti_3_C_2_T*_x_* heterostructure and comparative response behaviours of rGO/Ti_3_C_2_T*_x_*, MoS_2_/Ti_3_C_2_T*_x_*, and g−C_3_N_4_/Ti_3_C_2_T*_x_* towards multiple gases [96].

Graphene and its derivatives have been widely studied for their potential in detecting volatile organic compounds (VOCs) and humidity due to their high surface area, porosity, and thermal stability. Liu et al. [104] developed a VOC sensor at room temperature using a CuO/Ti_3_C_2_T_x_/rGO composite. The porous networks, high surface areas, uniform CuO dispersion, and high electron conductivity of the composite led to acetone sensing at room temperature with excellent sensing properties. The sensor showed a response of 52% to 100 ppm acetone gas with a quick response/recovery time (6.5 s/7.5 s) and exceptional selectivity and reproducibility. This approach using 3D rGO/MXene structures with MOS offers a new way for the development of room-temperature VOC sensors. Wang et al. [105] reported a room-temperature formaldehyde (HCHO) sensor using a rGO/N-Ti_3_C_2_T_x_/TiO_2_ composite. The rGO sheets acted as an excellent conduction stage for charge carriers in wet conditions, while the layered N-Ti_3_C_2_T_x_ promoted the diffusion and adsorption of formaldehyde and H_2_O molecules [105]. TiO_2_ nanostructures provided more sorption spots and enabled the dissociation of adsorbed water. Lee et al. [106] analysed the sensing behaviour of MXene/GO-fibres using the wet spinning technique as shown in Figure 8a. The synergetic effect of electronic attributes and adsorption of MXene and graphene allowed the fibres to display an NH_3_ response of 6.77% at room temperature. Song et al. [107] introduced a MOS-graphene-based MXene composite for the smart detection of NO_2_. The TiO_2_/rGO showed a sensitivity of 400% compared to pristine rGO due to the increased surface area and enhanced adsorption sites. The sensor displayed a very low detection limit of 50 ppb. The improved sensor properties were due to the homogeneous incorporation of MOS nanostructures in the rGO and the increased rGO interlayer distance initiated from the nanostructure intercalation [107]. Chachulia et al. [21] proposed a hydrogen sensor with a high response (159) for 1000 ppm of target gas at an operating temperature of 250 °C. MXene/polyurethane (PU) was introduced as an acetone sensor by Tang et al. [94] as a wearable VOC sensor. The microcracks formed in the sheath fibre could intensify the swelling due to transduction, resulting in an improved sensor response of 40% compared to the flat sheath fibre. Tran et al. [96] discussed a VOC sensor using a rGO/Ti_3_C_2_T_x_ composite for the detection of multiple harmful gases (NO_2_, CH_4_, and Toluene). The improved sensor performance of the composite was due to the active sites on the surface of the MXene and exceptional electron transfer across the MXene/rGO boundary [96] *(*Figure 8b).

**Table 3:** Comparison of various literature studies based on polymer/MXene and carbonaceous/MXene composite-based gas sensors.

Material	Target Gas	T	Response Time (s)	Recov. Time (s)	Conc.	Response (R_a_/R_g_) or (R_g_/R_a_)	Refs.
PANI/ Ti_3_C_2_T_x_	NH_3_	20 °C	-	-	50 ppm	400%	[98]
PANI/ MXene	C_2_H_6_O	RT	0.4 s	0.5 s	200 ppm	41.1%	[91]
Ti_3_C_2_T_x_/ PEDOT:PSS	CH_3_OH	RT	-	-	500 ppm	5.54	[93]
PEDOT:PSS/MXene	NH_3_	RT	116 s	40 s	(10 ppm), (1000 ppm)	9.6, 95	[97]
PVA/MXene	Humidity	-	0.9 s	6.3 s	11–97% RH	40	[99]
MXene/ chitosan	H_2_O	-	0.75 s	1.6 s	1–98 % RH	317	
MXene/ rGO/CuO	C_3_H_6_O	RT	6.5 s	7.5 s	100 ppm	52	[104]
rGO/nitrogen-doped MXene/ TiO_2_	HCHO	20 °C	-	-	4/20 ppm	26/132	[105]
MXene/ graphene	NH_3_	RT	-	-	100 ppm	7.21	[106]
MXene/ Poly ethyleneimine	CO_2_	20 °C	-	-	3000 ppm	~1	[101]
MXene-derived TiO_2_/rGO	NO_2_	RT	78 s	210 s	20 ppm.	165	[107]
							[21]
MWCNTs/ graphene/ MXene	H_2_	250 °C	-	-	1000 ppm	159.07	[94]
MXene/ polyurethane	-	-	-	-	-	-	[96]
rGO/ Ti_3_C_2_T_x_	VOC	RT	-	-	-	-	[102]
MXene/ TiO_2_/ cellulose	NH_3_	RT	76 s	62 s	100 ppm	6.84	[98]

### 5.4. MXene/Noble-Metal Heterostructures

Theoretical research has suggested that substitution of surface groups of MXenes with noble metals can potentially enhance the gas-sensing abilities of MXene/noble-metal compounds. The incorporation of noble metals brings about environmental resistance to corrosion and oxidation and contributes to the catalytic nature, thereby improving the sensor reactions. Additionally, noble metals act as electron traps, halting the rapid electron-hole recombination, which is beneficial to the sensor performance. Therefore, noble-metal functionalised MXenes represent a promising solution for improved sensing mechanisms, charge carriers, and VOC selectivity [108]. In this regard, Zhu et al. [53] proposed a room-temperature H_2_ sensor, utilising Pd-activated MXene nanosheets, which are lightweight and flexible. The sensor indicated a response time of 32 s and a sensitivity of 23 at 4% H_2_. Furthermore, Xu et al. [109] investigated the H_2_S sensing ability of Ag-incorporated MXene at room temperature and in a humid atmosphere. The study elucidates that the H_2_S-detection performance can be attributed to chemical and electronic sensitisation. The morphological images of the as-synthesised samples. The Ag/MXene sensor exhibited a wide range of detection from 0.05–10 ppm, a good LOD of 35 ppb, and fast response and recovery times of 34 s and 58 s, respectively. See Table 4 for the detailed sensing properties of MXene/Noble-Metal Heterostructures.

### 5.5. MXene/Metal Chalcogenide Heterostructures

**Figure 9:**
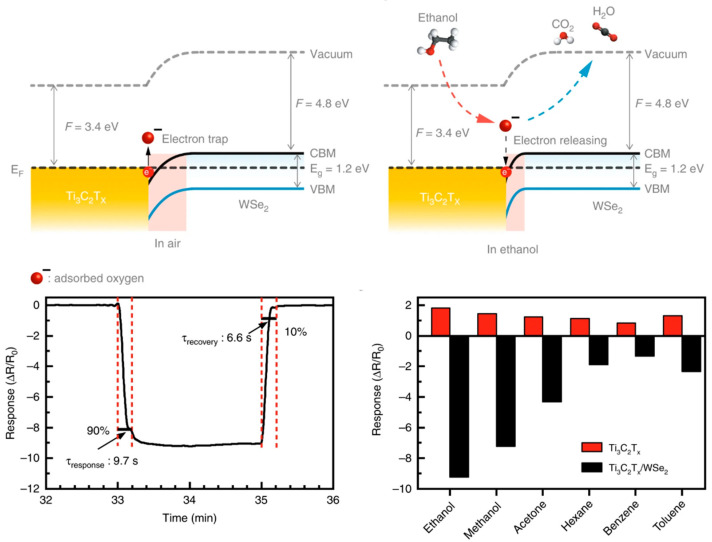
Energy-band diagram of the Ti_3_C_2_T*_x_*/WSe_2_ in air and ethanol, showing the variation of the depletion layer with interaction between adsorbed oxygen species and ethanol molecules. Response and recovery times calculated for 40 ppm of ethanol and selectivity test of the Ti_3_C_2_T*_x_* and Ti_3_C_2_T*_x_*/WSe_2_ sensors upon exposure to various VOCs at 40 ppm [110].

Out of various gas-sensing materials, metal chalcogenides are commonly used for the detection of different VOCs, but there is a lack of research on MXene/metal-chalcogenide composites for gas-sensing applications. Only a handful of articles in the literature highlight on MXene/metal-chalcogenide composites’ potential for detecting gases such as ethanol and nitrogen dioxide. Chen et al. [110] proposed a flexible ethanol sensor employing Ti_3_C_2_T_x_/WSe_2_, which demonstrated good reproducibility and repeatability. The band diagram showed an n-type response and modulation of the Schottky barrier, with the sensitivity being 11 times greater than that of pure MXene. Additionally, the dichalcogenide enhanced response and recovery times (9.7 s/6.6 s) compared to pure MXene. The work’s schematic is shown in Figure 9. Xia et al. [111] explored a room-temperature NO_2_ sensor with fast response and recovery times and excellent repeatability. Visible-light photoactivation improved the optoelectronic properties and NO_2_ sensing, while the heterointerface effectively separated photocarriers. See Table 4 for the detailed sensing properties of MXene/Metal Chalcogenide Heterostructures.

**Table 4:** Comparison of various literature studies based on noble-metals/chalcogenide/MXene composite-based gas sensors.

Material	Target Gas	T	Response Time (s)	Recov. Time (s)	Conc.	Response (R_a_/R_g_) or (R_g_/R_a_)	Refs.
MXene@Pd	H_2_	RT	-	-	-	40	[108]
Ag-MXene	H_2_S	-	34	58	10 ppm	-	[109]
Ti_3_C_2_T_x_/WSe_2_	C_2_H_6_O	RT	9.7	6.6	40 ppm	0.24	[110]
MXene/WS_2_	NO_2_	RT	56	53	10 ppb	-	[111]

## 6. Conclusions and Outlook

Continuing the discussion of the potential of MXene-based heterostructures for gas-sensing applications, it is important to note that these materials hold promise due to their unique properties that distinguish them from other gas-sensing materials. For example, MXenes possess high surface-to-volume ratios, which enable efficient gas adsorption and increase the chances of gas–molecule interactions. Additionally, the tuneable band gaps of MXenes allow for the detection of different gases through selective tuning of their electronic properties. However, despite these promising properties, there are still several challenges and limitations that need to be addressed to fully exploit the potential of MXene-based heterostructures for gas-sensing applications. One of the primary challenges is the degradation of MXenes when exposed to certain gases, which can affect their sensing performance. Therefore, there is a need to develop strategies to enhance the stability of MXenes in different gas environments to improve their sensing capabilities.

Another significant challenge is the development of highly sensitive and selective gas sensors using MXene-based heterostructures. Achieving this requires a deep understanding of how these materials interact with different gases. To accomplish this, it is vital to comprehend how MXene-based heterostructures interact with gases, which can be achieved by utilising the DFT computational method. This approach enables the accurate prediction of electronic and structural characteristics based on atomic and molecular configurations, making it an ideal tool for investigating gas sensing in MXenes. DFT calculations can also be used to simulate the adsorption of various gases on the surfaces of MXene-based heterostructures, providing insights into their gas-sensing performance and identifying the most promising materials for gas-sensing applications. Additionally, DFT calculations can evaluate the impact of functionalisation on the structural and electronic properties of MXene-based heterostructures, providing a roadmap for improving their gas-sensing capabilities. However, DFT calculations are limited to theoretical predictions and may not reflect the actual behaviour of MXene-based gas sensors in real-world environments. Therefore, it is essential to combine computational simulations with experimental investigations to verify the effectiveness of MXene-based heterostructures for gas sensing. In situ measurement techniques, such as gas chromatography, infrared spectroscopy, and mass spectrometry, can provide real-time information on the interaction between MXene-based heterostructures and gases. These techniques enable researchers to measure the gas concentration and determine the reaction kinetics and mechanisms of gas adsorption and desorption on MXenes’ surfaces. By combining theoretical simulations with experimental measurements, researchers can gain a comprehensive understanding of the gas-sensing mechanism of MXene-based heterostructures and identify the most promising materials for gas-sensing applications.

The economic feasibility and environmental assessment of MXene-based heterostructures for gas-sensing applications are important factors to consider in the development of these materials. One advantage of MXenes is that they are relatively low-cost materials, which makes them economically feasible for large-scale production. However, the cost of synthesising MXene-based heterostructures can increase significantly due to the additional processing steps required to create the heterostructures. Therefore, cost-effective fabrication techniques should be developed to reduce the overall cost of production. In terms of environmental assessment, MXenes have several advantages over other gas-sensing materials. MXenes are highly stable and do not degrade easily when exposed to different gases, which reduces the need for frequent replacement and disposal of sensors. Additionally, MXenes can be synthesised from abundant and environmentally friendly sources, which makes them more sustainable compared to other materials that require rare or toxic elements. However, the environmental impact of the fabrication processes for MXene-based heterostructures should be carefully evaluated to ensure that they do not pose any risks to the environment or human health. Strategies such as green synthesis techniques and efficient waste management practices should be implemented to minimise the environmental footprint of MXene-based heterostructures.

In conclusion, MXene-based heterostructures hold great promise for gas-sensing applications due to their unique properties. However, there are still several technical challenges and limitations that must be addressed, including improving the stability of MXenes in different gas environments and developing highly sensitive and selective gas sensors using these materials. In addition to technical challenges, the economic feasibility and environmental impact of MXene-based gas sensors must also be considered. The large-scale synthesis of MXene-based heterostructures requires cost-effective and environmentally friendly synthesis routes. Additionally, the disposal of MXenes after use must be handled in an environmentally sustainable manner. Therefore, researchers must work towards developing scalable and sustainable synthesis routes and explore environmentally friendly methods for the disposal of MXenes. Despite these challenges, the potential applications of MXene-based heterostructures in gas sensing are vast, ranging from environmental monitoring to medical diagnostics. The continued exploration and optimisation of these materials for gas-sensing applications can lead to the development of highly sensitive, selective, and reliable gas sensors, ultimately contributing to the advancement of various industries and the betterment of society as a whole.

## Data Availability

Not applicable.
